# Superior tumor cell killing by oxygen ion beam irradiation compared to carbon ions and photons in pancreatic Cancer cells – An in vitro investigation

**DOI:** 10.1016/j.ctro.2026.101225

**Published:** 2026-06-30

**Authors:** Lixin Mai, Aleksei Smirnov, Michael M. Allers, Zhengyang Song, Muzi Liu, Thuy Trinh, Stephan Brons, Fabian Weykamp, Jakob Liermann, Ramon Lopez Perez, Jürgen Debus, Peter E. Huber, Jonathan M. Schneeweiss

**Affiliations:** a Division of Molecular and Radiation Oncology, German Cancer Research Center (DKFZ), Heidelberg, Germany; b Department of Radiation Oncology, Heidelberg University Hospital, Medical Faculty Heidelberg, Heidelberg University, Heidelberg, Germany; c Heidelberg Ion Therapy Center (HIT), Department of Radiation Oncology, Heidelberg University Hospital, Heidelberg, Germany; d Heidelberg Institute for Radiation Oncology (HIRO), Heidelberg, Germany; e Clinical Cooperation Unit Radiation Oncology, DKFZ, Heidelberg, Germany

**Keywords:** Pancreatic cancer, Radiotherapy, Oxygen-ion radiation, Carbon-ion radiation, Gemcitabine, DNA damage

## Abstract

Pancreatic cancer remains one of the most treatment-refractory malignancies, displaying profound resistance to chemotherapy and radiotherapy. While the benefit of photon radiotherapy remains debated, heavy-ion modalities such as carbon ions show promise in overcoming radioresistance. Here, we extend this paradigm by evaluating oxygen-ion beam irradiation in pancreatic cancer cells and directly comparing its efficacy with carbon ions and photons. Oxygen ions induce higher clonogenic cell killing in human pancreatic cancer cells, with a relative biological effectiveness (RBE) around two times higher for oxygen ions than for carbon ions. Moreover, their growth-inhibitory effects are substantially less dependent on gemcitabine-mediated radiosensitization than those of carbon ions or photons. Mechanistically, oxygen-ion irradiation induces greater direct and complex DNA damage, slows down DNA repair kinetics, and augments apoptosis following combined gemcitabine treatment, accompanied by sustained activation of DNA damage response pathways. These findings delineate the distinct radiobiological advantages of oxygen ion beam irradiation in pancreatic cancer and highlight their potential as a powerful modality to overcome radioresistance, providing a compelling rationale for further translational and clinical exploration.

## Highlights

• ^16^O-RT reduces greater pancreatic cancer clonogenic survival than ^12^C-RT) and Ph-RT by enhancing DNA damage and apoptosis.
• The greater clonogenic survival reduction of ^16^O-RT is less dependent on gemcitabine-based radiosensitization.
• ^16^O-RT upregulates and sustains DNA damage repair pathway activity by inducing ATM phosphorylation.
• ^16^O-RT may be a promising heavy-ion radiotherapy modality in overcoming the radioresistance of pancreatic cancer.

## 1 Introduction

Pancreatic cancer (PC) remains one of the most lethal malignancies, with survival rates among the lowest across cancer types [1]. Two major factors drive these poor outcomes: the typically asymptomatic nature of early disease, resulting in late-stage diagnosis and limited eligibility for curative surgery [2], [3]; and the profound therapeutic resistance of advanced pancreatic cancer to chemotherapy, immunotherapy, and radiotherapy (RT) [3], [4], [5], [6]. For patients with locally advanced pancreatic cancer (LAPC), the standard therapies include FOLFIRINOX [7] or gemcitabine-based chemotherapy regimens with or without radiotherapy. These approaches provide modest benefit, delaying disease progression by only a few months [8], [9], [10]. Improved therapeutic strategies are urgently needed.

Charged-particle radiotherapy has emerged as a promising avenue, and Proton therapy has shown encouraging results in neoadjuvant [11], concurrent [12], [13] and adjuvant [14] settings. However, carbon ions exhibit considerably higher LET and RBE than protons. While the RBE of both particle types is influenced by LET and tends to increase towards the distal region of the Bragg peak, the effect is substantially more pronounced for carbon ions [15]. Consequently, carbon ion therapy may provide biological advantages for the treatment of radioresistant and hypoxic tumors while maintaining favorable normal tissue sparing [16], [17], [18]. Accordingly, early clinical studies combining carbon-ion irradiation with gemcitabine have demonstrated feasibility and potential benefit in LAPC [19], [20]. Despite these advances, the optimal irradiation strategy for PC remains undetermined. More recently, preclinical data on helium ions have further expanded the interest in alternative particle modalities [21], while the potential of oxygen ion beam irradiation—a heavier ion with potentially higher RBE—has been largely unexplored in PC. In particular, the radiobiological and therapeutic properties of oxygen-ion beams—especially their relative biological effectiveness, mechanisms of DNA damage and repair, and interactions with standard chemotherapeutics such as gemcitabine—have not been systematically examined for pancreatic cancer.

Given the hypoxic and intrinsically radioresistant nature of pancreatic tumors [22], [23], [24], oxygen ions may provide distinct advantages. To clarify their therapeutic potential, we investigate the functional and mechanistic effects of oxygen-ion irradiation in pancreatic cancer cells in vitro and directly compare them with carbon-ion and photon irradiation, both alone and in combination with gemcitabine, the current standard chemotherapeutic agent. We show that oxygen-ion beams exert stronger tumor cell killing effects than carbon ions or photons and show a reduced dependence on gemcitabine-mediated radiosensitization. Oxygen ions induce more extensive and complex DNA damage, slow repair kinetics, and enhance apoptosis after combined treatment, accompanied by sustained activation of DNA damage response pathways. These findings highlight the radiobiological advantages of oxygen ions and support their development as a promising strategy to overcome radioresistance in pancreatic cancer.

## 2 Material and methods

### 2.1 Cell culture

The cell lines PANC-1 (#CRL-1469) and BxPC-3 (#CRL-1687) were purchased from the American Type Culture Collection (ATCC) and cultured according to the recommended provider's guidelines. Both cell lines were cultured in RPMI-1640 medium supplemented with 10% fetal bovine serum, 2 mM l-Glutamine and 2 mM Penicillin/Streptomycin at 37 °C, 5% CO_2_.

### 2.2 Irradiation

Photon irradiation was performed using a MultiRad 225 irradiation system (Precision X-Ray). A tube voltage of 200 kV and a tube current of 17.8 mA resulted in a final dose rate of 2.151 Gy/min. A 2 mm copper filter was used for hardening the beam. Carbon ion and oxygen ion beam irradiation were performed at the Heavy Ion Therapy Center (HIT), Heidelberg, Germany. ^16^O-ions and ^12^C-ions were used with specific beam energies of 143.6–162.9 MeV/u (range in water: 34.3–43.4 mm) and 120.4–138.7 MeV/u (range in water 33.4–43.4 mm), respectively. These beams were used to create a spread-out Bragg peak (SOBP) with a 10 mm width in rasterscan mode at a horizontal beam line. During irradiation, samples were positioned at a depth of 35 mm within the SOBP. At this depth, the dose-averaged linear energy transfer (LETd) values were 145 keV/μm and 92 keV/μm for the oxygen and carbon beams, respectively. All experiments were carried out at room temperature under normoxic conditions. Doses of 1, 3, 6, 9 Gy (photons), and 0.5, 1, 2, 3 Gy (^12^C-ions and ^16^O-ions) were applied. Supplement Fig. S1 provides dose and LETd profiles of the carbon and oxygen treatment plans for a 1 Gy irradiation dose.

### 2.3 Colony formation assay

Colony formation assays were performed as described earlier [25], [26]. For combined treatment experiments, cells were treated with respective doses (10 nM, 50 nM) of gemcitabine (Gemcitabine-HEXAL®) for 4 h before irradiation. Survival curves were fitted according to the linear-quadratic model using GraphPad Prism software (version 10.6). Two to three biologically independent experiments were performed with technical triplicates. Details are described in the Supplements.

### 2.4 Flow cytometry

Cells were harvested and washed with PBS, fixed with 3% paraformaldehyde (PFA) for 10 min and permeabilized with 70% ethanol. Intracellular staining was performed with Alexa Fluor 647-labelled anti-active Caspase-3 antibody (1:20 dilution; BD #560626), AF488-labelled anti-γH2AX antibody (1:20; Biolegend #613406) and 4′,6-Diamidin-2-phenylindol (DAPI) (1:100) as described previously [27]. Around 10000 cells per sample were acquired using an LSR Fortessa (BD Bioscience) flow cytometer. Data analysis was performed using FlowJo software (version 10.10.1), Microsoft Excel and GraphPad Prism (version 10.6).

### 2.5 Fluorescence microscopy

20 μl of cell suspensions (cells stained as described above) were applied onto glass cover slips, dried at room temperature and embedded onto glass slides using Flouromount G. Subsequently, images were captured using a Zeiss Axioplan 2 fluorescence microscope (Zeiss, Oberkochen, Germany) and MetaCyte Metafer v.4 software. Afterwards, TIFF images were analyzed with MATLAB and Microsoft Excel. Manual quality control of processed images was performed randomly across samples to ensure appropriate micronuclei identification. Around 500 nuclei per sample were investigated whenever possible.

### 2.6 Western blotting

Western Blotting was performed as described earlier [25], [26], [28]. Primary antibodies against p-ATM (R&D, #MAB22902), BRCA1 (Cell signaling, #9010), and β-actin (Cell Signaling Technology, #4967) were used, followed by washing with TBS-T and incubation with horse radish peroxidase-linked secondary antibodies (Cell Signaling Technology, #7074, #7076). Protein bands were detected with an Amersham 680 Imager and quantified with ImageJ software (version 1.54f). Details are described in the Supplements.

### 2.7 Resazurin viability assay

The resazurin cell viability assay was performed according to the manufacturer's instructions, in biological triplicates. Details are in the Supplements.

### 2.8 Statistics

Data analysis was performed using Microsoft Excel, R software (version 4.4.0) and GraphPad Prism software (version 10.6). Clonogenic survival curves were compared via Beam-pairwise Extra sum-of-squares F test. For other data sets one-way or two-way ANOVA with subsequent Šídák's or Dunnett's multiple comparisons testing was applied, as detailed in the figure legends.

## 3 Results

### 3.1 Oxygen ion beam irradiation exhibits greater clonogenic survival reduction compared to carbon ion beam and photon irradiation in pancreatic cancer cells

**Fig. 1:**
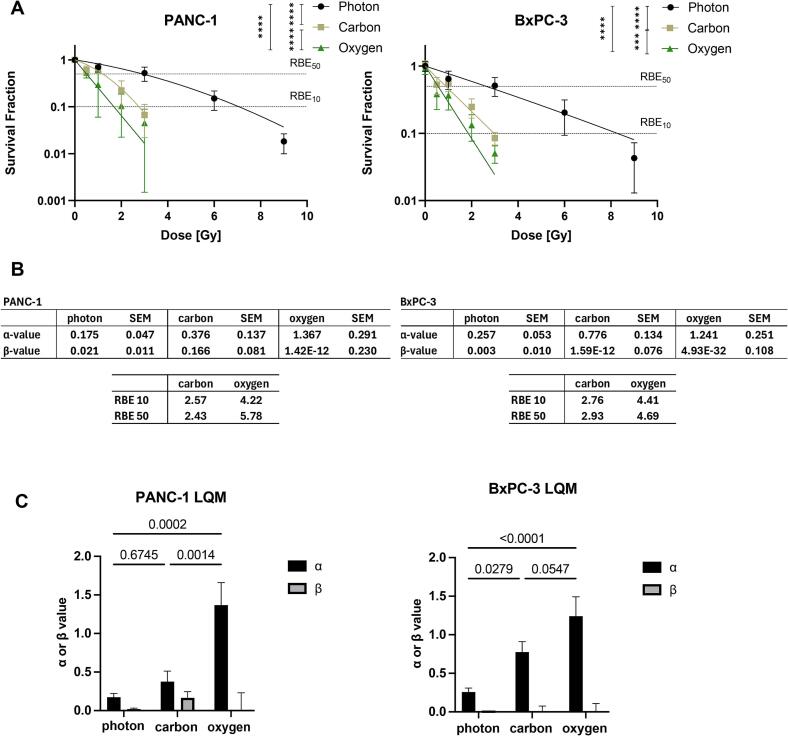
Oxygen irradiation exhibits enhanced anti-tumor efficacy compared to carbon ion beam and photon irradiation in pancreatic cancer cells. (A) PANC-1 and BxPC-3 were irradiated with different doses of photon, carbon ion or oxygen ion beam and formation of colonies was assessed 7 to 10 days after radiotherapy (RT). Survival curves were fitted according to the linear-quadratic model (LQM). Data are derived from *n* = 3 independent experiments with 3 technical replicates each for each cell line (mean ± SD). Beam-pairwise Extra sum-of-squares F test was used for statistical analysis of differences between beam-type induced survival curves. (B) α and β values and relative biological effectiveness (RBE) values (RBE10 and RBE50) compared to photons were calculated thereafter. (C) Representation and statistical analysis of differences between α and β LQM values (mean ± SEM) between different beam types. 2-way ANOVA with subsequent Sidak's multiple comparisons test. *P < 0.05, ***P < 0.001, ****P < 0.0001; ns, not significant.

To evaluate the impact on clonogenic survival of oxygen-ion irradiation compared to carbon ions and determine corresponding RBE values, we assessed clonogenic survival in human PANC-1 and BxPC-3 pancreatic cancer cells following exposure to oxygen ion beam radiotherapy (^1^⁶O-RT), carbon ion beam radiotherapy (^12^C-RT), or photon radiotherapy (Ph-RT). In both cell lines, ^1^⁶O-RT induced the strongest reduction in surviving fractions at the investigated physical doses, indicating the highest cytotoxic potency among all modalities (Fig. 1A).

Relative biological effectiveness (RBE) values at 10% survival were substantially higher for oxygen ions than for carbon ions (PANC-1: 4.2 vs. 2.6; BxPC-3: 4.4 vs. 2.8), and this pattern was even more pronounced at 50% survival (PANC-1: 5.8 vs. 2.4; BxPC-3: 4.4 vs. 2.9) (Fig. 1B). Whereas RBE₁₀ and RBE₅₀ values were similar after ^12^C-RT, ^1^⁶O-RT produced consistently higher RBE₅₀ values, reflecting a less pronounced repair shoulder thus indicating stronger repair reduction by oxygen ions compared to carbon ions [29].

In the linear–quadratic radiobiological model [30], the fitted α value represents the dose-proportional (linear) component of radiation-induced cell killing. Consistent with the above findings about RBE, α-values derived from survival curves were significantly higher for ^1^⁶O-RT than for ^12^C-RT or Ph-RT in both cell lines (Fig. 1C). Together, these results suggest that oxygen ions exert enhanced cellular clonogenic survival reduction compared with carbon ions and photons in pancreatic cancer cells in vitro.

### 3.2 Oxygen-ion irradiation maintains greater clonogenic survival reduction compared with photons after combined treatment with gemcitabine

**Fig. 2:**
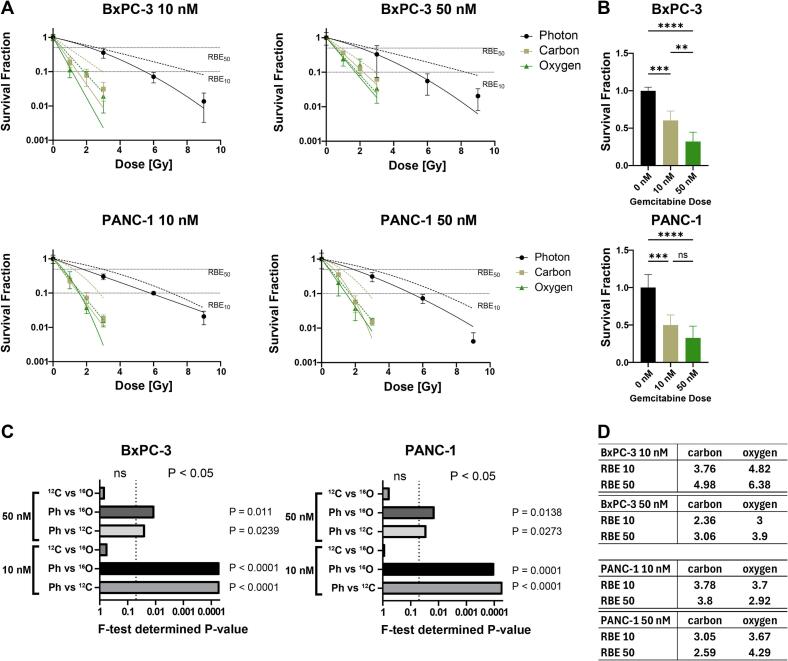
Gemcitabine sensitizes pancreatic cancer cells to photon and carbon ion radiation, but shows less synergism with oxygen ion radiation. A) BxPC-3 and PANC-1 cells were treated with 50 nM and 10 nM vs. 0 nM (dotted line) gemcitabine and subsequently irradiated with different doses of photon, ^12^C-ion or ^16^O-ion beam vs. unirradiated controls. Formation of colonies was assessed 7 to 10 days after RT. Survival curves were fitted according to the linear-quadratic model (LQM) and compared by beam-pairwise Extra sum-of-squares F test. Dashed lines represent survival curves of RT only groups (see Fig. 1 for measured data points) and solid lines represent the combined treatments. All data are normalized to 0 Gy plus respective dose of gemcitabine. B) Survival fractions in BxPC-3 and PANC-1 cells after gemcitabine treatment. Statistical analysis was performed with ordinary one-way ANOVA and post hoc Sidak's for multiple comparisons. C) Representation of inter-beam pairwise F-test determined *P*-values for beam-modality survival curve comparisons after respective gemcitabine treatment. D) Relative biological effectiveness (RBE) values (RBE_10_ and RBE_50_) after combined treatment compared to the corresponding combined photon plus gemcitabine dose group. Data are derived from *n* = 2 independent experiments with 3 technical replicates each. *P < 0.05, **P < 0.01, ***P < 0.001, ****P < 0.0001; ns, not significant.

Gemcitabine is widely used as a radiosensitizer in combined chemoradiotherapy for locally advanced pancreatic cancer [9], [31], [32]. In pancreatic cancer cells, gemcitabine alone reduced clonogenic survival in a dose-dependent manner (Fig. S2), resulting in approximately 60% survival at 10 nM and 30% survival at 50 nM (Fig. 2B). Importantly, both 10 nM and 50 nM gemcitabine induced pronounced radiosensitization—defined as a synergistic, over-additive reduction in clonogenic survival—after photon and carbon-ion irradiation, with a stronger effect observed for photons than for carbon ions. In contrast, the combination of gemcitabine with oxygen-ion irradiation resulted predominantly in additive effects only (Fig. 2A, S3A). Despite the weaker radiosensitization, carbon ions and also oxygen ions retained substantially greater anti-tumor efficacy than photons when combined with gemcitabine (Fig. 2C, D). The reduced radiosensitizing effect of gemcitabine with heavy-ion beams resulted in similar clonogenic survival profiles for carbon- and oxygen-ion irradiation (Fig. 2C).

Similarly, RBE values at 10% and 50% survival were higher in oxygen ion beams-treated groups than those treated with carbon ion beams in both tested gemcitabine concentrations (Fig. 2D), except the comparison in PANC-1 treated with low concentration gemcitabine (10 nM) (RBE_10_ of ^16^O vs. ^12^C: 3.7 vs. 3.78; RBE_50_: 2.92 vs. 3.8). Because combined survival fractions were calculated relative to Gem-treated, unirradiated controls to assess synergy, absolute cell-killing effects reflect the added impact of both treatments. These findings indicate that the intrinsic, high cytotoxic potency of heavy ions, particularly ^16^O-RT, minimizes the need for synergistic radio-sensitization by chemotherapy.

### 3.3 Oxygen ion irradiation induces enlarged DNA-damage foci and delayed repair kinetics, resulting in persistent DNA damage

To assess how different radiation modalities affect DNA damage and repair dynamics, we quantified γH2AX foci formation—an established surrogate for DNA double-strand breaks—using radiation doses that were approximately biologically equivalent based on clonogenic survival.

**Fig. 3:**
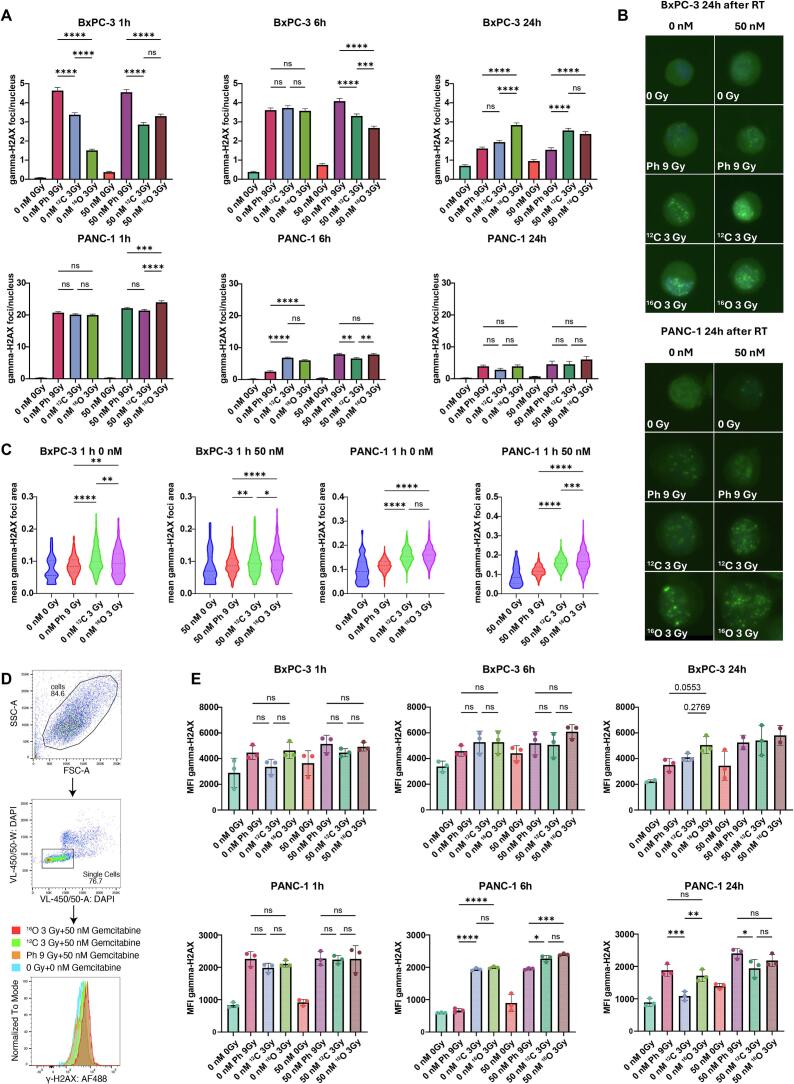
Oxygen ion radiation attenuates DNA damage repair and induces persistent DNA double-strand breaks. BxPC-3 and PANC-1 cells were treated with 0 or 50 nM gemcitabine and subsequently irradiated with 9 Gy photons, 3 Gy ^12^C-ions, 3 Gy ^16^O-ions or mock-irradiated. The samples were fixed at 1 h, 6 h and 24 h after irradiation and DNA double-strand breaks were evaluated by immunofluorescence microscopy (A-C) and flow cytometry (D-E) of γH2AX. A) Number of γH2AX foci per nucleus (mean ± SEM). B) Representative images of γH2AX foci at 24 h after RT. C) Violin plots of the average γH2AX foci sizes per nucleus at 1 h after RT. D) Gating strategy for flow-cytometry based assessment of E) γH2AX median fluorescence intensity (MFI) in BxPC-3 and PANC-1 cells 1 h, 6 h and 24 h after RT. Data are derived from n = 3 replicates for all experiments. Data are mean ± SD. Statistical analysis was performed with ordinary one-way ANOVA and post hoc Sidak's multiple comparisons. *P < 0.05, **P < 0.01, ***P < 0.001, ****P < 0.0001; ns, not significant.

Immunofluorescence microscopy revealed robust γH2AX induction across all modalities in PANC-1 and BxPC-3 cells (Fig. 3A, B). Although initial γH2AX foci numbers were higher after photon irradiation, both ^1^⁶O and ^12^C ions generated significantly larger γH2AX foci at 1 h post-irradiation (*P* < 0.0001) (Fig. 3C), consistent with more complex DNA lesions. Overall, γH2AX signal intensity at this early time point did not differ between beam types (Fig. S4). Following combination treatment, gemcitabine plus ^1^⁶O irradiation induced larger foci than gemcitabine plus ^12^C (Fig. 3B, C).

The enlarged foci induced by ^1^⁶O ions were most pronounced at early time points but remained evident up to 24 h post-irradiation (Fig. 3A, B, S5). Notably, γH2AX foci numbers after ^1^⁶O and ^12^C irradiation surpassed photon-induced foci numbers at later time points (6–24 h), indicating persistent DNA lesions and attenuated repair (Fig. 3A). In gemcitabine-treated PANC-1 cells, elevated γH2AX foci after ^1^⁶O irradiation persisted for at least 72 h (Fig. S6).

Flow cytometry (FC) analysis corroborated these findings, demonstrating sustained increases in γH2AX median fluorescence intensity (MFI)—particularly after ^1^⁶O irradiation—most prominently at 6 h and 24 h for PANC-1 and BxPC-3 cells, respectively (Fig. 3D, E). Overall, ^16^O-RT induces a biologically distinct and more complex DNA damage profile than carbon ions and photons that leads to compromised repair, supporting its enhanced cytotoxic efficacy in pancreatic cancer cells.

### 3.4 Oxygen beam irradiation induces enhanced apoptosis and more complex micronuclear damage

**Fig. 4:**
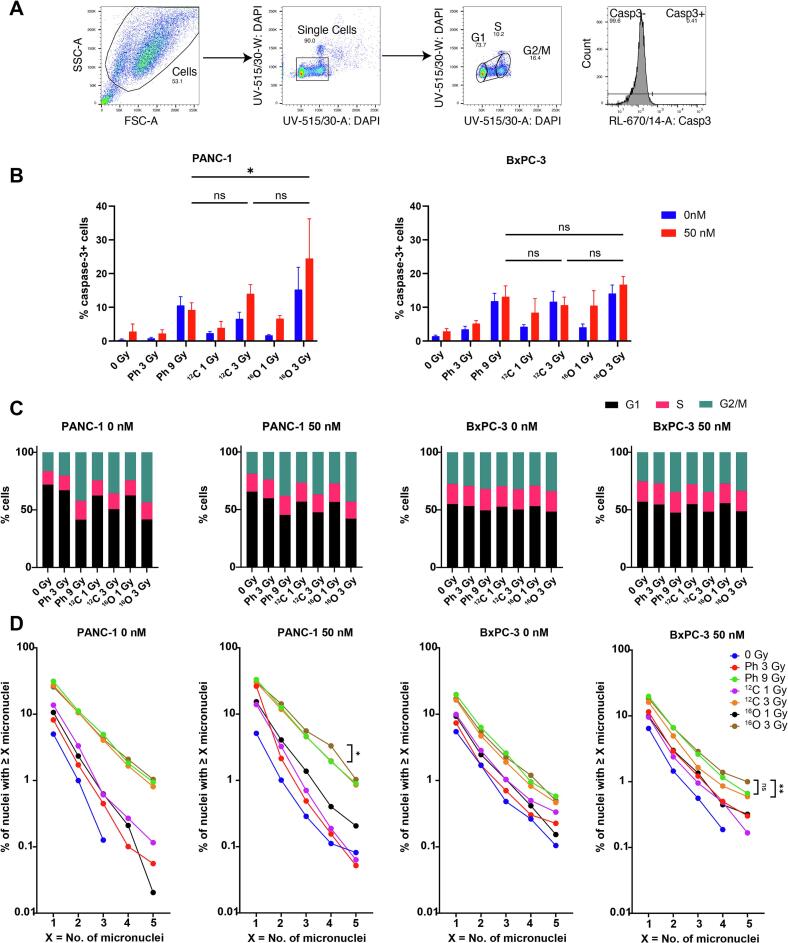
Oxygen ion irradiation induces enhanced apoptosis rates and more complex DNA-damage patterns in pancreatic cancer cells after combination with gemcitabine. BxPC-3 and PANC-1 cells were treated with 0, 10 or 50 nM gemcitabine and subsequently irradiated with photons (3 or 9 Gy), ^12^C-ions (1 or 3 Gy) or ^16^O-ions (1 or 3 Gy). Non-irradiated samples were included as controls. Samples were fixed at 72 h after irradiation and assessed by flow cytometry. A) Gating strategy, B) Apoptosis (caspase 3 activation). C) Cell cycle arrest (DAPI intensity). D) Percentage of nuclei with ≥ indicated amount of micronuclei assessed by fluorescence microscopy. Manual quality control of processed images was performed randomly across samples to ensure appropriate micronuclei identification. Around 500 nuclei per sample were investigated whenever possible. Data are derived from n = 3 independent experiments. Data in B) are mean ± SD. Statistical analysis was performed using ordinary one-way ANOVA with Sidak's post hoc multiple-comparisons test. *P < 0.05, **P < 0.01, ***P < 0.001, ****P < 0.0001; ns, not significant.

We next examined how ^1^⁶O-RT affects cell cycle dynamics and apoptosis in PANC-1 and BxPC-3 cells using flow cytometry (Fig. 4A). At 72 h after treatment, gemcitabine combined with ^1^⁶O-RT induced significantly higher apoptosis rates in PANC-1 cells compared with Gem-Ph-RT, with a similar though not statistically significant trend in BxPC-3 cells (Fig. 4B). Sub-G1 DNA fragmentation showed comparable patterns (Fig. S7). At 24 h, early apoptosis and sub-G1 fractions were low across all modalities (Fig. S8A, B), but a greater proportion of cells accumulated in G₂/M phase after ^1^⁶O-RT and ^12^C-RT relative to photons in both cell lines (Fig. S8C). By 72 h, cell cycle distributions had largely equalized between modalities, although a dose-dependent G₂ accumulation persisted (Fig. 4C).

We also quantified micronuclei (MN) formation by immunofluorescence microscopy in both cell lines (Fig. 4D, S9). Higher radiation doses increased the number of MN-positive nuclei for all modalities. Importantly, heavy ions caused substantially more micronucleated cells than photons, and also the number of cells with multiple micronuclei was higher for heavy ions, indicating more complex DNA damage. For example, the percentage of BxPC-3 cells with ≥5 MN per nucleus was significantly higher for Gem–^16^O-RT versus Gem–Ph-RT (*P* = 0.0073) and the same was observed for PANC-1 cells with ≥4 MN per nucleus (*P* = 0.0434) (Fig. 4D).

### 3.5 Oxygen ion beam irradiation induces prolonged DNA-damage repair pathway activity

**Fig. 5:**
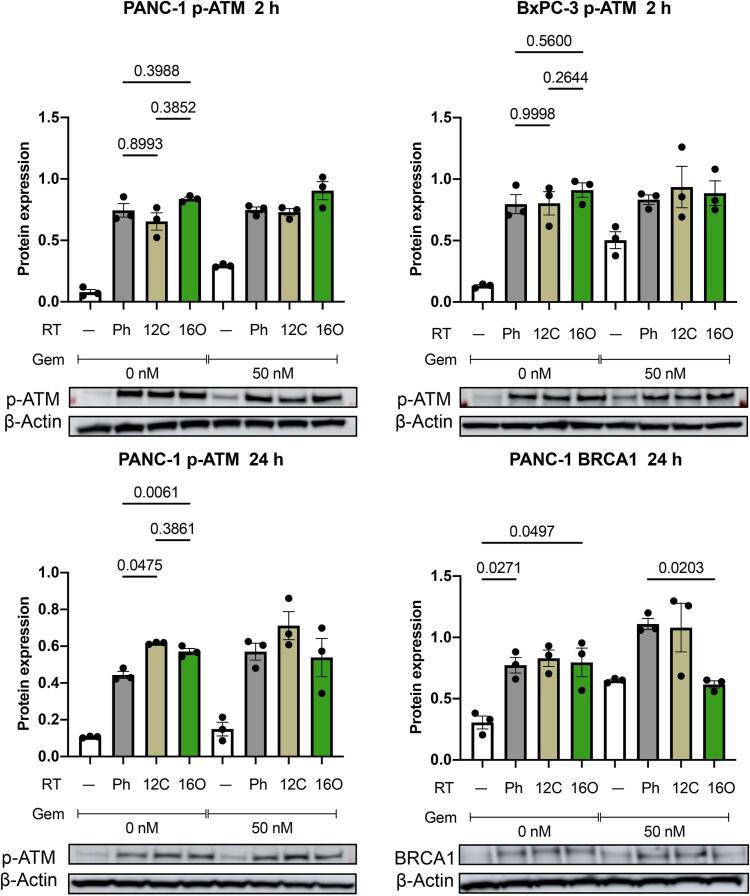
Prolonged Persistence of DNA Damage Repair Responses after Oxygen-Ion Beam Irradiation Compared to Photons. PANC-1 and BxPC-3 cells were treated with 50 nM vs. 0 nM gemcitabine and subsequently irradiated with 9 Gy Photon, 3 Gy ^12^C-ion or 3 Gy ^16^O-ion beam vs. unirradiated controls. 2 h and 24 h after RT, protein lysates were collected for subsequent western blotting. A) Quantification of relative protein expression of indicated markers at 2 h and 24 h after RT. All lanes were corrected for their corresponding loading control (beta-actin) after membrane stripping. B) Representative western blot images of investigated proteins. Data are mean ± SD from n = 3 replicates. Statistical analysis was performed using ordinary one-way ANOVA followed by Sidak's post hoc multiple-comparisons test. *P < 0.05, **P < 0.01; ns, not significant.

We next examined activation of the Ataxia Telangiectasia Mutated (ATM) kinase, the master regulator of the cellular response to DNA double-strand breaks (DSBs) [33]. All beam modalities induced robust phosphorylation of ATM (p-ATM) 2 h after irradiation in BxPC-3 and PANC-1 cells (Fig. 5A). Although not statistically significant, ^16^O irradiation produced the highest p-ATM levels at this early time point. Gemcitabine alone induced p-ATM to approximately 50% of the radiation-induced levels, but its combination with any radiation type did not further enhance ATM phosphorylation.

At 24 h post-irradiation, gemcitabine-induced p-ATM upregulation had subsided, whereas all radiation modalities continued to show sustained p-ATM elevation. Charged particles induced significantly higher p-ATM levels than photons (^16^O-RT: *P* = 0.0061; ^12^C-RT: *P* = 0.0475), while oxygen and carbon ions did not differ significantly from each other (Fig. 5B). As observed at 2 h, gemcitabine did not alter p-ATM levels in combination with any radiation type at 24 h.

Because ATM phosphorylation indicates activation of the DNA damage–repair (DDR) response, we next assessed the expression of the homologous recombination (HR)–associated protein BRCA1 [34], [35]. All radiation modalities increased BRCA1 expression 24 h after irradiation, with no statistically significant differences between beam types. Gemcitabine alone also induced BRCA1 to approximately 50% of radiation-induced levels. Notably, gemcitabine further increased BRCA1 expression after photon and carbon irradiation, but this enhancement was not observed following oxygen-ion irradiation. This absence of a combined effect with ^16^O-RT parallels to the findings for ATM phosphorylation (Fig. 5C).

## 4 Discussion

Here we show in in vitro experiments using pancreatic cancer cells that oxygen ion irradiation enhanced cellular clonogenic survival reduction compared to conventional photon radiotherapy and carbon ions. The enhanced efficacy of oxygen ions is accompanied by reduced dependence on gemcitabine-mediated radiosensitization, establishing a highly effective intrinsic cytotoxic mechanism. Mechanistically, we found that oxygen-ion irradiation produces greater direct and complex DNA damage, which subsequently delays repair and enhances apoptosis, all in the context of a prolonged activation of DNA damage response pathways. Conclusively, these data reveal distinct radiobiological advantages of oxygen ions, supporting their promise for potentially overcoming pancreatic cancer radioresistance. However, this will be of significance only if less pronounced adverse effects occur in normal tissue.

Radiotherapy is a cornerstone of cancer treatment. Conventional radiotherapy uses high-energy photon beams (typically 6 MV) generated by medical linear accelerators. Although charged-particle radiotherapy was first explored as early as the 1930s [36], only proton therapy has achieved substantial clinical adoption. Compared with photons, all charged particles—including protons and heavier ions—offer superior dose conformity due to their distinct physical interactions with tissue, culminating in a Bragg peak.

Heavier ions such as carbon, helium, and oxygen provide an additional potential benefit: increased biological effectiveness at equal physical doses. Currently, carbon ions are the most commonly used ions for heavy-ion beam irradiation in clinical practice. Carbon ions exhibit approximately 2–3-fold greater cell-killing effectiveness (relative biological effectiveness, RBE) than photons or protons. Early clinical experiences from 1975 to 1992 in Berkeley, CA [37] were followed by systematic clinical programs in Japan in 1994 [38] and in Darmstadt / Heidelberg, Germany in 1997 [39] and 2009 [22], [40], [41], [42], respectively.

Today, heavy-ion therapy is available at a limited number of centers. Clinical carbon-ion data exist for several tumor entities, including pancreatic cancer series [20], [43], [44]. Helium-ion therapy has only recently re-entered clinical use as a lighter alternative to carbon (or heavier alternative to protons) [45], and experimental data in pancreatic cancer remain sparse [21]. Another potential heavy ion, oxygen ion, may be considered as a heavier alternative to carbon. Since oxygen ions have a higher charge, they exhibit higher LET than carbon ions and therefore may provide a better biological profile, such as more pronounced DNA damage [46] and decreased oxygen dependence [16], [47], [48]. However, evidence for oxygen-ion therapy is even more limited in pancreatic cancer. Although clinical reports for pancreatic cancer are yet unavailable, treatment with oxygen (and neon ions) has already commenced at QST, Japan, for head and neck cancers [49], [50] and bone and soft tissue tumors [51]. Compared with carbon ions, oxygen ions possess a higher nuclear charge (Z = 8 versus Z = 6), resulting in increased LET and denser ionization tracks that may contribute to the induction of more complex DNA damage. However, RBE does not increase with LET forever; at some point towards higher LET the RBE goes down again due to overkill effects [52].

In Bedford's framework [30] of lethal, sub-lethal, and potentially lethal DNA damage within the linear-quadratic model of clonogenic survival, the α-component (linear term) reflects irreparable, single-event lethal lesions, while the β-component (quadratic term) reflects repair and misrepair of sub-lethal damage. In our pancreatic cancer models, ^1^⁶O irradiation resulted in the highest α-component compared with ^12^C and photons and a higher RBE compared with ^12^C (4.2–5.8 for ^1^⁶O vs. 2.4–2.9 for ^12^C), suggesting a greater induction of complex, directly lethal DNA lesions. Increased complexity of carbon ion–induced DNA damage relative to photons has been shown previously [25], [53], including in pancreatic cancer. Thus, our findings indicate that oxygen ions enhance this effect further. In contrast, the β-components of the survival curves did not substantially differ among the beam modalities, indicating that the higher efficacy of ^1^⁶O-RT compared with ^12^C-RT is driven primarily by increased induction of complex, directly lethal DNA lesions. This suggests that ^1^⁶O-RT may help overcome the pronounced radioresistance of pancreatic cancer, partially caused by its hypoxic tumor microenvironment [24], because more direct lethal DNA damage is associated with lower oxygen-enhancement ratios. [22]. Accordingly, recent clinical studies have reported encouraging local control with ^12^C-RT in locally advanced pancreatic cancer [54]. Our data, therefore suggest that ^1^⁶O-RT could further improve local tumor control relative to carbon ions in future clinical investigations.

Gemcitabine is the guideline-recommended agent for combination with surgery or radiotherapy in advanced pancreatic cancer. We found that gemcitabine administered before irradiation showed radiosensitization to Ph-RT and ^12^C-RT, whereas it did not significantly alter survival curves after ^1^⁶O-RT, indicating an absence of radiosensitization for ^1^⁶O-RT. Potential mechanisms of gemcitabine in carbon ion and photon radiosensitization include effects of Gemcitabine to induce S-phase arrest, deplete dNTP pools, and promote misrepair of DNA [55]. These effects may enhance radiotherapy effects in particular under conditions of replication stress [31]. However, previous studies have reported only minimal or no substantial radiosensitization when chemotherapy is combined with ^12^C-RT compared with photon radiotherapy across several tumor types, including pancreatic cancer [56], [57], [58]. Likewise, only marginal additional effects were observed for the Gem–^12^C-RT and Gem–^1^⁶O-RT combinations compared with ^1^⁶O-RT alone, suggesting a strong intrinsic efficacy of ^1^⁶O-RT as a monotherapy. This may be attributable to the pronounced G2/M cell-cycle arrest induced by ^1^⁶O-RT, which could diminish the additional impact of gemcitabine-mediated interference with DNA damage repair. The more extensive and complex DNA damage induced by oxygen ion radiotherapy may limit the additional benefit of gemcitabine-mediated radiosensitization. However, this interpretation warrants further experimental validation.

Our analysis of DNA-damage foci formation and repair kinetics further supports the notion that ^1^⁶O-RT induces more complex, directly lethal DNA lesions. High-LETd radiation, such as carbon ions, generates larger and structurally more complex γH2AX foci than low-LETd photon irradiation, characterized by subfoci clustering and prolonged persistence of DNA damage [53], [59], [60], [61], [62]. Accordingly, we observed that ^1^⁶O-RT produced larger and more intense γH2AX foci compared with ^12^C-RT and photons. γH2AX signals appeared as early as 1 h post-irradiation, peaked at 24 h, and remained elevated beyond 24 h, indicating a higher burden of clustered, complex DNA double-strand breaks. The larger foci induced by ^1^⁶O-RT were also accompanied by increased numbers of γH2AX foci at later time points, suggesting less efficient DNA repair.

We also investigated ATM-kinase activation as a key mediator of cellular DNA-repair responses [63]. Consistently, all beam modalities induced strong ATM phosphorylation at 2 h after RT. Notably, persistently enhanced ATM-phosphorylation after ^16^O-RT and ^12^C-RT compared to Ph-RT reflected the prolonged persistence of DNA-DSB and attenuated DNA damage repair kinetics in pancreatic cancer cells [33].

Moreover, the observed high directly lethal effectiveness of ^16^O-RT in pancreatic cancer might function via the HR repair pathway: The HR-marker BRCA1 exhibits rapidly increased protein levels as a response to RT-induced DNA-damage [64], while severe DNA damage induces proteasomal BRCA1-degradation with subsequent induction of cellular apoptosis [65]. Here, we found that with the combination of gemcitabine, ^16^O-RT reduced intracellular BRCA1 levels compared to Ph-RT, and subsequently enhanced apoptosis rates were observed. This high intracellular BRCA1 consumption with subsequent enhanced apoptosis induction may reflect the complex and persistent DNA-damage after ^16^O-RT [35], [65].

However, it is worth noticing that in this study, due to the physical properties of oxygen ions, higher LETd levels were employed in oxygen ion beams than in carbon ion beams (around 145 keV/μm (range 125–175 keV/μm) for oxygen ions versus 92 keV/μm (range 84–102 keV/μm) for carbon ions). In our experimental study, much higher LETd levels were used compared to those typically achieved in clinical practice. Based on previous research, the minimum LETd, the most critical parameter for tumor control, applied in conventional ^12^C-RT to pancreatic cancer is around 40–50 keV/μm [66], [67], or slightly higher reaching 70–80 keV/μm at most with the advanced techniques such as LETd optimization and arc therapy [68]. Concerning ^1^⁶O-RT, less data is reported except for a clinical study optimizing the treatment plan for multi-ion therapy in head and neck tumors, where a minimum LETd of about 100 keV/μm is described with LETd optimization [49]. Therefore, the data of our preclinical research experiments should be interpreted cautiously, considering the selected parameters including the specifically investigated LETd levels.

Overall, our results suggest that the high, directly lethal anti-tumor effectiveness of ^1^⁶O-RT is largely independent of cell-cycle state [22], and chemotherapy-induced radiosensitization. Consequently, ^1^⁶O-RT may retain its therapeutic potency in vivo irrespective of vascularization, oxygenation, hypoxia or immune response. Hypoxia is a major resistance mechanism for conventional radiotherapy with photons. Therefore, ^1^⁶O-RT might be especially beneficial for tumors in which the delivery of systemic therapies is limited by biological barriers or tumors that are considered immunologically cold [69], [70], [71]. Radiotherapy-induced lymphopenia has been reported to be lower after ^12^C-RT compared to Ph-RT. While this effect can at least partially be explained by reduced irradiated volumes, the improved overall survival [72] implicitly suggested the involvement of the immune system in the overall treatment effect. For example, we and others had previously highlighted the role of macrophage repolarization and the vascular system in the treatment of pancreatic tumors using low-dose photon radiation [73] and also identified a role of NK cells in the tumor response to photon radiotherapy [28]. Additionally, we had shown that photon, proton and carbon ion radiation induced directional lymphocyte migration towards pancreatic cancer [26]. We have not investigated immune cells or used in vivo tumor models in mice in the current study, which is another distinct limitation, but also offers suggestions for further research. However, in the present study, we found that oxygen ions induced more complex DNA-damage patterns in BxPC-3 and PANC-1 pancreatic cancer cells, accompanied by more micronuclei (MN) per nucleus. Elevated MN formation has been associated with activation of the cGAS–STING pathway and promoting anti-tumor immune responses [74], which may suggest enhanced antitumor immune response by ^16^O-RT versus conventional radiotherapy.

## 5 Conclusion

This study suggests oxygen-ion irradiation (^16^O-RT) as a potentially promising heavy-ion modality for the radiotherapy treatment of pancreatic cancer. ^16^O-RT showed superior anti-tumor efficacy and induced more direct DNA damage than photons and carbon ions in human pancreatic cancer cells under the investigated experimental LETd levels. This was evidenced by enlarged and persistent γH2AX foci and prolonged activation of DNA damage–repair pathways. In addition, ^1^⁶O-RT increased apoptosis in combination with chemotherapy and generated more complex micronuclei patterns. Together, these effects suggest that ^16^O-RT may overcome key mechanisms of pancreatic cancer radioresistance, including hypoxia and low tumor immunogenicity. Further in vivo studies and clinical trials integrating assessment of normal tissue toxicity are warranted to fully explore the therapeutic potential of ^16^O-RT in pancreatic cancer.

## Declaration of generative AI use

Generative AI and AI-assisted technologies were NOT used in the preparation of this work.

## CRediT authorship contribution statement

**Lixin Mai:** Writing – review & editing, Writing – original draft, Visualization, Validation, Methodology, Investigation, Formal analysis, Data curation, Conceptualization. **Aleksei Smirnov:** Writing – review & editing, Validation, Methodology, Investigation. **Michael M. Allers:** Writing – review & editing, Validation, Methodology, Conceptualization. **Zhengyang Song:** Validation, Methodology, Investigation. **Muzi Liu:** Methodology, Investigation, Formal analysis. **Thuy Trinh:** Validation, Methodology, Investigation. **Stephan Brons:** Writing – review & editing, Validation, Methodology, Investigation. **Fabian Weykamp:** Writing – review & editing. **Jakob Liermann:** Writing – review & editing. **Ramon Lopez Perez:** Writing – review & editing, Software, Methodology. **Jürgen Debus:** Writing – review & editing, Resources. **Peter E. Huber:** Writing – review & editing, Writing – original draft, Supervision, Resources, Project administration, Data curation. **Jonathan M. Schneeweiss:** Writing – review & editing, Writing – original draft, Visualization, Validation, Supervision, Project administration, Formal analysis, Data curation, Conceptualization.

## Funding

This study was partially supported by the Overseas Study Program of Guangzhou Elite Project (Guangzhou, China) to LXM, and by the Mildred Scheel Program of Deutsche Krebshilfe e.V. (German Cancer Aid) to MMA and JMS.

## Declaration of competing interest

The authors declare that they have no known competing financial interests or personal relationships that could have appeared to influence the work reported in this paper.
