# Supplementary material for: Superior tumor cell killing by oxygen ion beam irradiation compared to carbon ions and photons in pancreatic Cancer cells – An in vitro investigation
Source: Clin Transl Radiat Oncol. 2026 Jun 30;60:101225. doi: 10.1016/j.ctro.2026.101225 (PMC13355022; doi:10.1016/j.ctro.2026.101225)
Supplement: Supplementary file 1 — Supplementary material [file mmc1.docx]

**Supplements to**

**Superior Tumor Cell Killing by Oxygen Ion Beam Irradiation Compared to Carbon Ions and Photons in Pancreatic Cancer Cells – An In Vitro Investigation**

Lixin Mai, Aleksei Smirnov, Michael M. Allers, Zhengyang Song, Muzi Liu, Thuy Trinh, Stephan Brons, Fabian Weykamp, Jakob Liermann, Ramon Lopez Perez, Jürgen Debus, Peter E. Huber, Jonathan M. Schneeweiss

**Supplementary Methods**

**Colony Formation assay**

For combined treatment experiments, cells were treated with respective doses (10nM, 50nM) of gemcitabine (Gemcitabine-HEXAL®) for 4 hours before irradiation and subsequently incubated in fresh medium for 7 to 10 days at 37°C, 5 % CO_2_ for colony formation. Subsequently, cells were fixed with 33% acetic acid in methanol for 10 minutes and stained with 0.1% crystal violet staining solution (2 g Crystal violet +10% ethanol/L ddH_2_O). Colonies (> 50 cells) were counted manually. Survival fractions were calculated according to the formula SF (x Gy) = [PE (x Gy)] ⁄ [PE (0 Gy)] = [seeded cells x Gy / mean counted colonies x Gy]/[ seeded cells 0 Gy / mean counted colonies 0 Gy]. Survival curves were fitted according to the linear-quadratic model using GraphPad Prism software (version 10.6). All experiments were performed two to three times independently with technical triplicates for each round.

**Western Blotting**

Cells were lysed in radio-immunoprecipitation (RIPA) buffer with additional protease and phosphatase inhibitors (Invitrogen). Protein concentrations were measured with a BCA kit (Thermo Fisher, #23227). 5-10 µg of protein per lane were separated on 3-8% Tris-acetate acrylamide gels (Invitrogen) or 10% handcast Tris-glycine acrylamide gels and afterwards blotted onto polyvinylidene difluoride (PDVF) membranes. Unspecific binding sites were blocked with 5% bovine serum albumin (BSA) and the membranes were incubated with primary antibodies against p-ATM (R&D, #MAB22902), BRCA1 (Cell signaling, #9010), β-actin (Cell Signaling Technology, #4967), followed by washing with TBS-T and incubation with horse radish peroxidase-linked secondary antibodies (Cell Signaling Technology, #7074, #7076). After incubation of the membranes with LumiGlo chemiluminescence reagent (Cell Signaling Technology, #40806655), protein bands were detected with an Amersham 680 Imager and quantified with ImageJ software (version 1.54f).

**Resazurin viability assay**

Cells were incubated with indicated doses of gemcitabine for 4 hours. Subsequently, medium was changed and cells were incubated for 3 to 5 days at 37°C, 5 % CO_2_. Afterwards, 250 µl of fresh medium and 50 µl resazurin solution (150 µg/ml, pH 7.4, 0.22 µm filtered) were added to all wells including medium only control groups for baseline fluorescence signal correction. After 2 hours of incubation, resofurin fluorescence was measured using a SpectraMax iD3 microplate reader (Ex./Em. 530/590 nm). All experiments were performed in biological triplicates.

**Fig. S1 Dose and dose-averaged linear energy transfer (LETd) distribution for carbon ion and oxygen ion beam irradiation.** Dose profiles (Gy) and LETd profiles (keV/µm) of 1 Gy carbon and 1 Gy oxygen ion beam are displayed as measured at the heavy ion beam therapy center (HIT) Heidelberg, Germany. For biological experiments, cell samples were placed at z = 35 mm to ensure irradiation within the ~10 mm-wide SOPB.


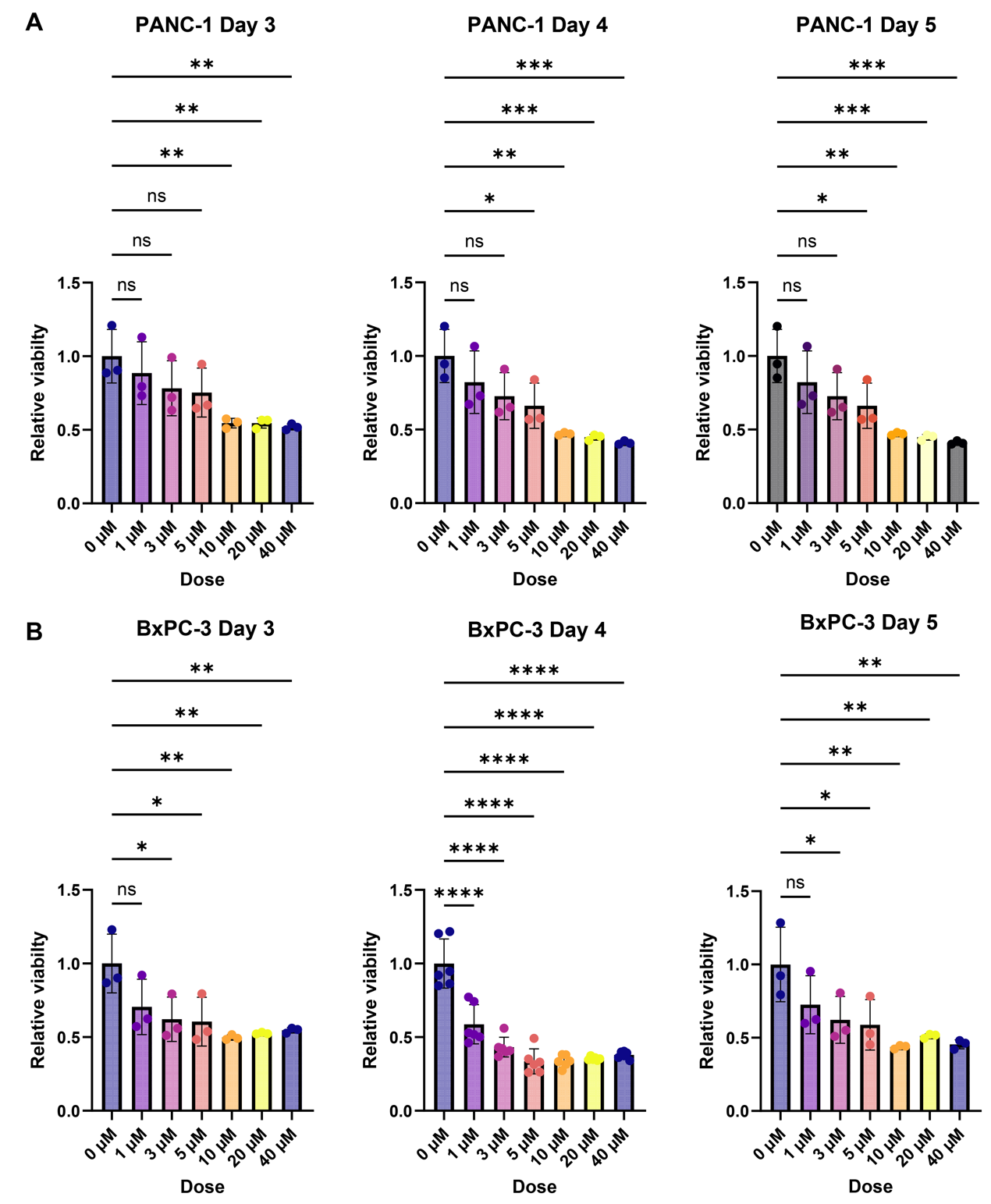


**Fig. S2 Dose-effect of gemcitabine on cell viability in pancreatic cancer cells.** The cellular viability of A) PANC-1 and B) BxPC-3 was assessed at the indicated time points after treatment with different doses of gemcitabine via the resazurin assay. Statistical analysis was performed using ordinary one-way ANOVA with Dunnett’s post hoc multiple comparisons test. *P < 0.05, **P < 0.01, ***P < 0.001, ****P < 0.0001; ns, not significant.


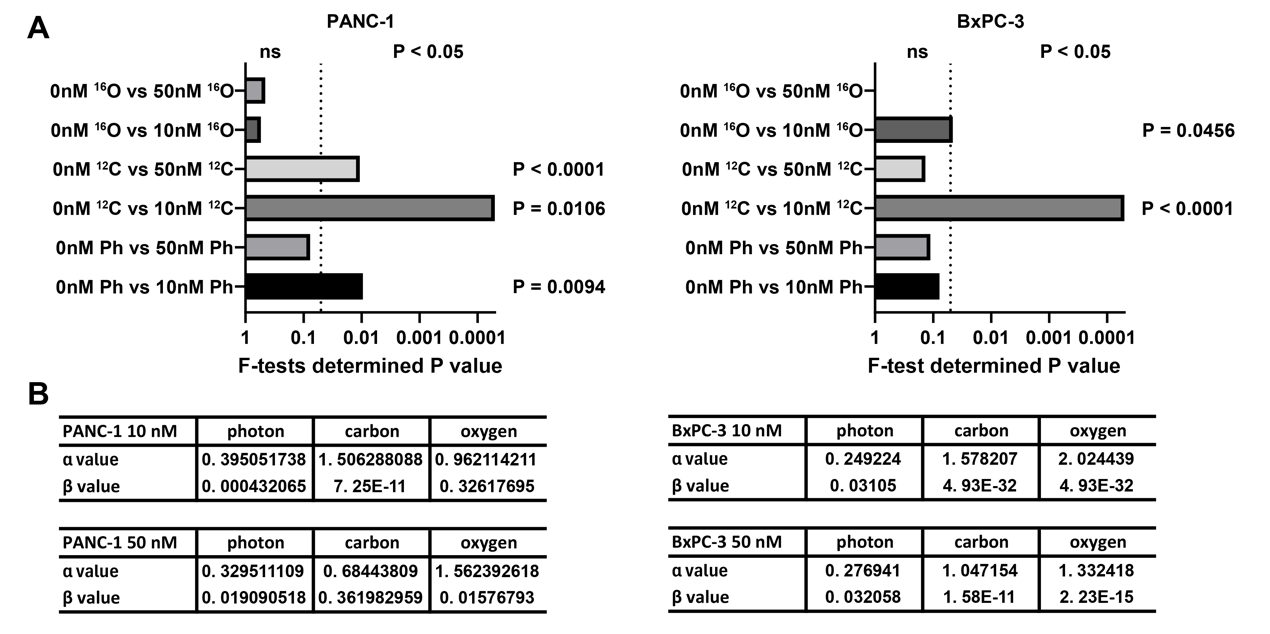


**Fig. S3 Radiosensitization effects of gemcitabine for each irradiation beam modality in PANC-1 and BxPC-3 cells.** A) Illustration of intra-beam pairwise F-test determined P-values for survival curve comparisons after treatment with different gemcitabine doses before RT application with the same beam-modality. All survival curves were normalized towards each 0 Gy plus the respective dose of gemcitabine treatment group. B) α & β values of survival curves fitted according to the linear quadratic model (LQM) for combined gemcitabine plus RT treatment colony formation assays. Data are derived from n = 2 independent experiments with n = 3 technical replicates each for each cell line.


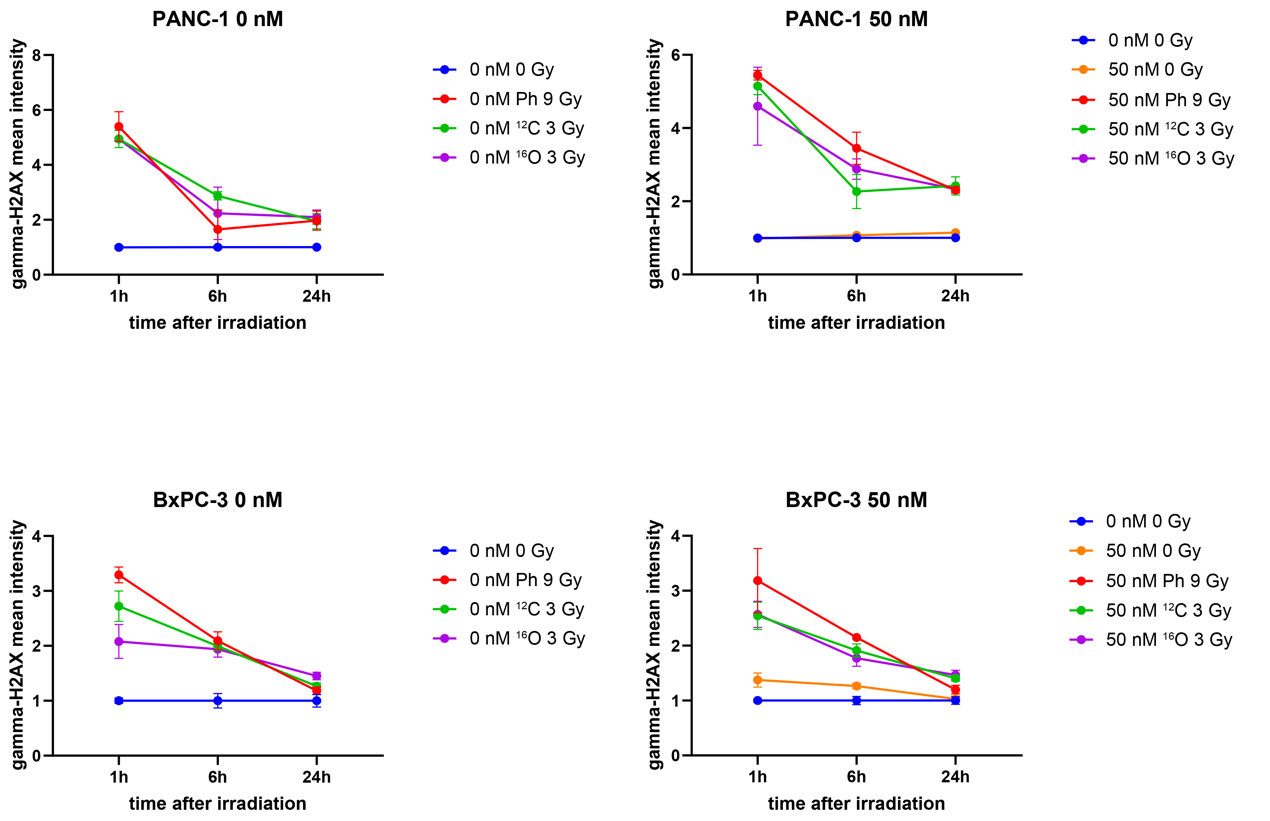


**Fig. S4 Mean γH2AX fluorescence intensity over time after irradiation in PANC-1 and BxPC-3 cells.** The mean γH2AX intensity within the nucleus was measured by microscopy at 1 h, 6 h and 24 h after irradiation and 0 nM or 50 nM gemcitabine treatment in BxPC-3 and PANC-1 cells. Data are mean ± SD from n = 3 replicates.


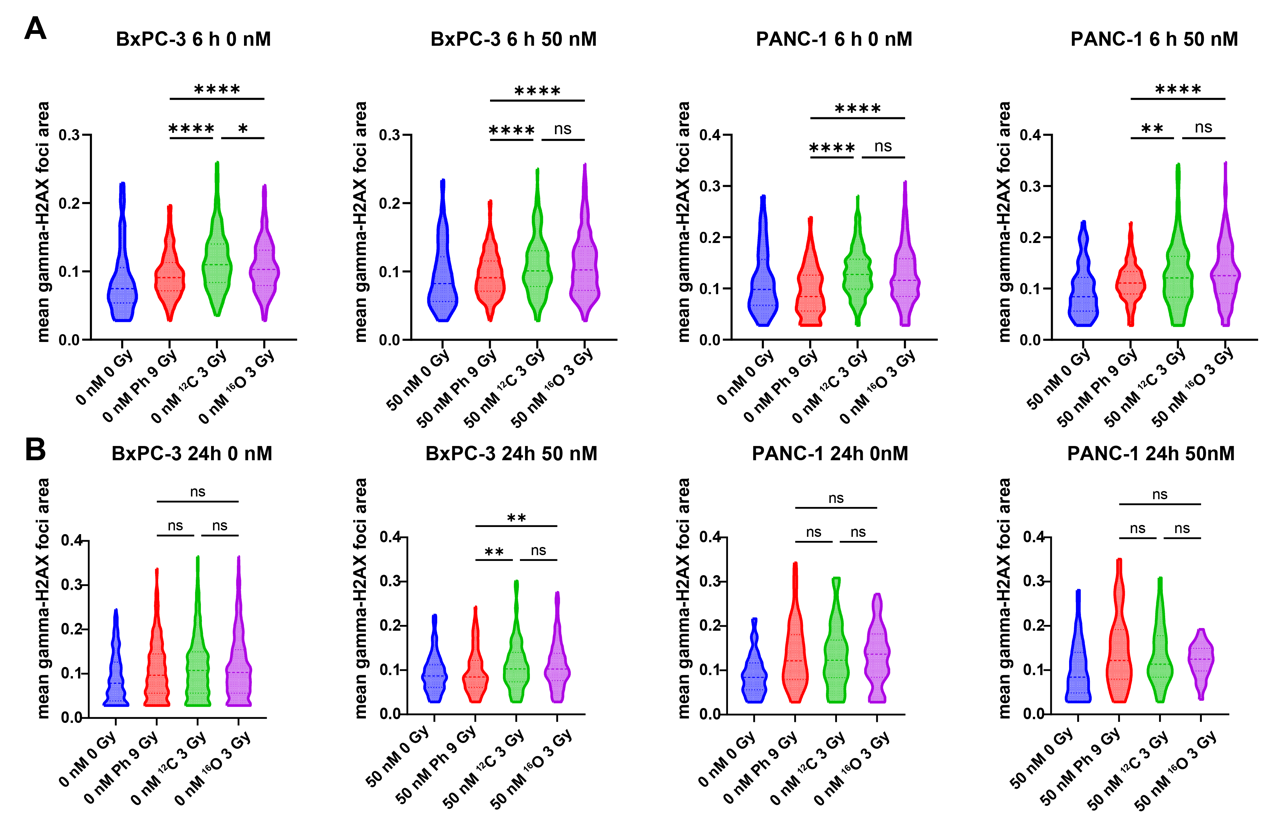


**Fig. S5 Average γH2AX foci area at 6 h and 24 h after irradiation in PANC-1 and BxPC-3 cells.** Violin plots of the average γH2AX foci sizes per nucleus at A) 6 h and B) 24 h after irradiation. Data are derived from n = 3 replicates for all experiments. Statistical analysis was performed using ordinary one-way ANOVA and post hoc Sidak’s multiple comparisons test. *P < 0.05, **P < 0.01, ***P < 0.001, ****P < 0.0001; ns, not significant. For quality control of automatically acquired microscopy images, outliers have been identified statistically via the ROUT-method (Q = 1%) equally for all groups.


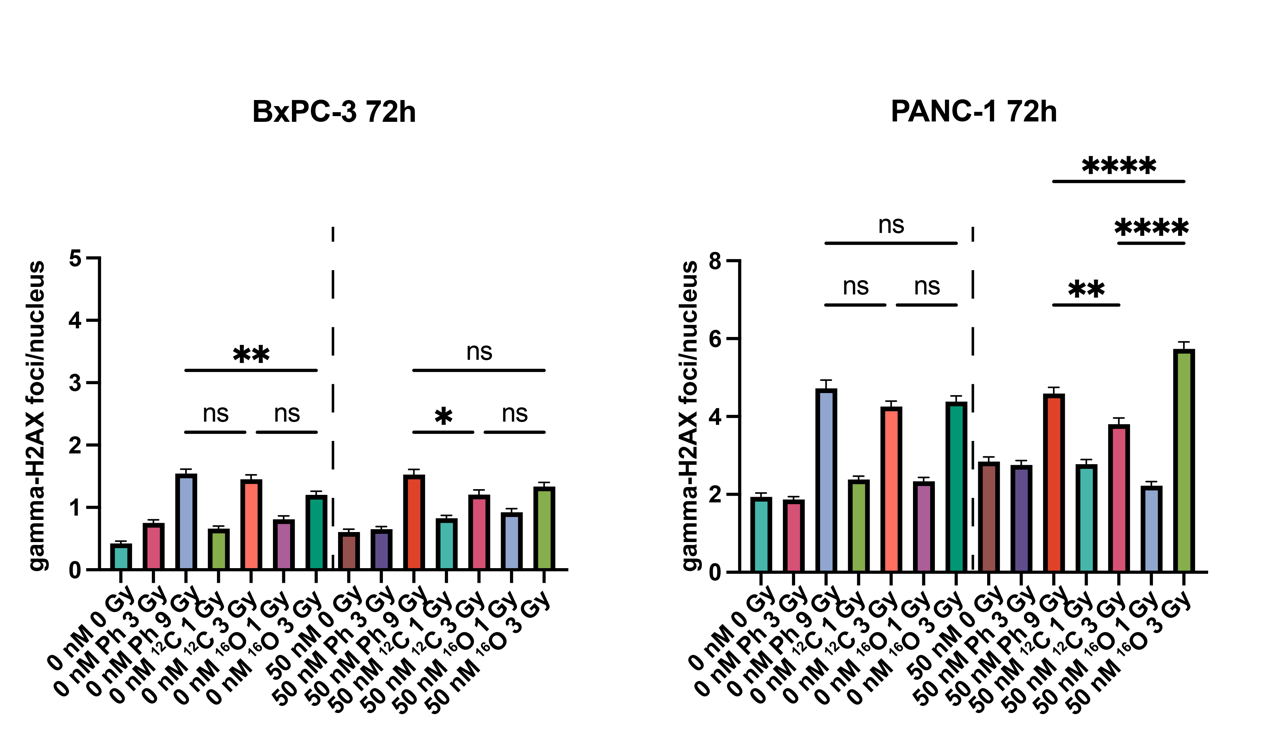


**Fig. S6 Number of γH2AX foci at 72 h after irradiation in PANC-1 and BxPC-3 cells.** The average number of γH2AX foci per nucleus were measured by immunofluorescence microscopy at 72 h after Ph-RT, ¹²C-RT or ¹⁶O-RT as monotherapy or in combination with 50 nM gemcitabine. Data are mean ± SEM from n = 3 independent experiments. Statistical analysis was performed using ordinary one-way ANOVA and post hoc Sidak’s multiple comparisons test. *P < 0.05, **P < 0.01, ****P < 0.0001; ns, not significant.


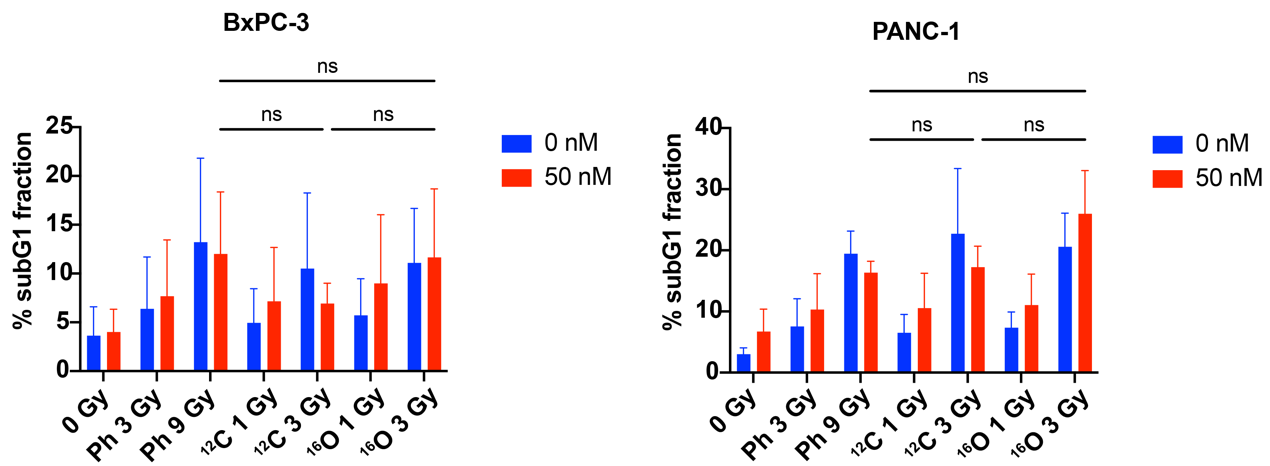


**Fig. S7 SubG1-fractions in BxPC-3 and PANC-1 cells at 72 h after irradiation.** Flow cytometry-based assessment of subG1-fraction as a percentage of total cell count in PANC-1 and BxPC-3 cells at 72 h after being treated with 50 nM vs 0 nM gemcitabine followed by irradiation with photons (3 Gy, 9 Gy), carbon ions (1 Gy, 3 Gy), or oxygen ions (1 Gy, 3 Gy) with unirradiated controls. Data are mean ± SD from n = 3 independent experiments. Statistical analysis was performed using ordinary two-way ANOVA and post hoc Sidak’s multiple comparisons test. ns, not significant.


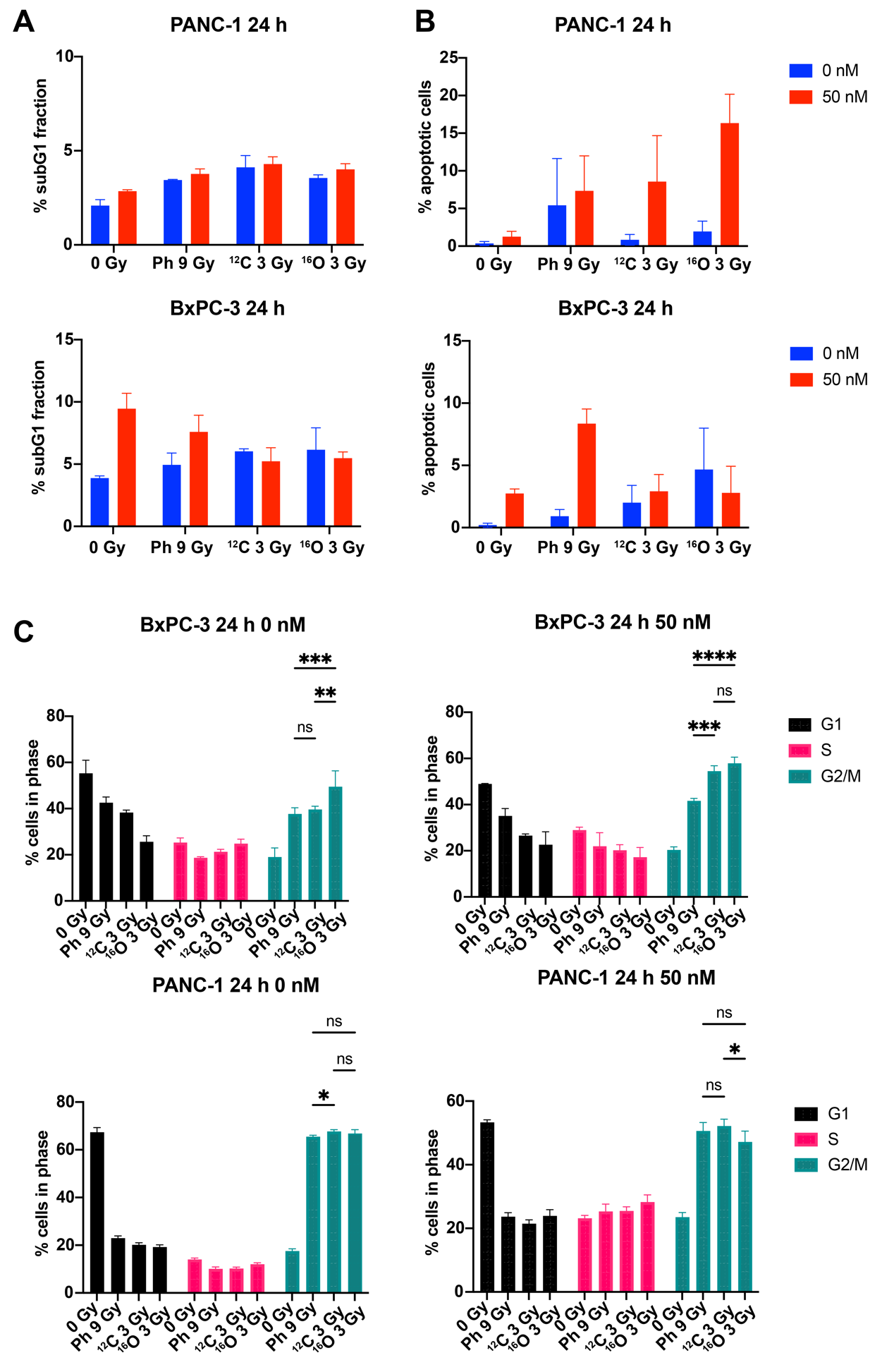


**Fig. S8 Apoptosis and cell cycle distribution in PANC-1 and BxPC-3 cells at 24 h after irradiation with photons, carbon ions or oxygen ions preceded by 50 nM or 0 nM gemcitabine treatment.** A) Flow cytometry (FC) -based assessment of subG1-fraction as percentage of total cell count in PANC-1 and BxPC-3 cells at 24 h after respective treatment. B) FC-based assessment of treatment-induced Caspase3 Alexa Fluor 647-positive apoptosis induction and C) FC-DAPI-based cell cycle (G1-phase, S-phase, G2/M-phase) analysis in PANC-1 and BxPC-3 cells. Data derived from n = 3 replicates for all experiments. Data are mean ± SD. Statistical analysis was performed using ordinary two-way ANOVA and post hoc Sidak’s multiple comparisons test. *P < 0.05, **P < 0.01, ***P < 0.001, ****P < 0.0001; ns, not significant.


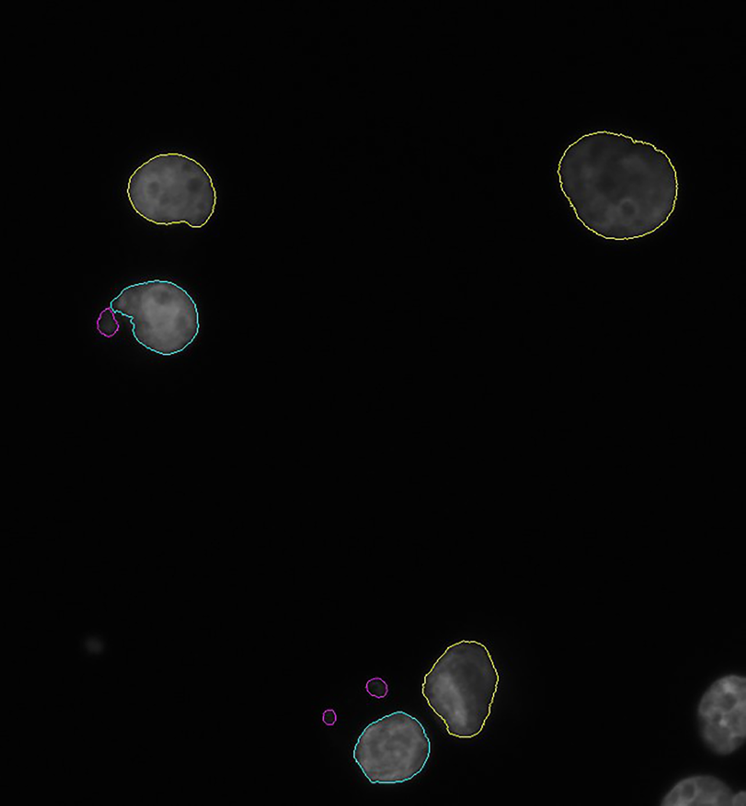


**Fig. S9 Micronuclei analysis in pancreatic cancer cells.** Micronuclei (MN, pink circles) were detected, assigned to their corresponding nuclei (cyan outline) and quantified in DAPI images using own algorithms implemented in MATLAB, as previously described [1]. Nuclei without MN are outlined in yellow.

References

[1] S. Regnery, H. Franke, T. Held, T. Trinh, A. Naveh, Y. Abraham, J. Horner-Rieber, J. Hess, P.E. Huber, J. Debus, R. Lopez Perez, S. Adeberg, Tumor treating fields as novel combination partner in the multimodal treatment of head and neck cancer, Head Neck, 45 (2023) 838-848.
